# Efficient and Reusable Iron Catalyst to Convert CO_2_ into Valuable Cyclic Carbonates

**DOI:** 10.3390/molecules26041089

**Published:** 2021-02-19

**Authors:** Ana P. C. Ribeiro, Peter Goodrich, Luísa M. D. R. S. Martins

**Affiliations:** 1 Centro de Química Estrutural and Departamento de Engenharia Química, Instituto Superior Técnico, Universidade de Lisboa, Av. Rovisco Pais, 1049-001 Lisboa, Portugal; apribeiro@tecnico.ulisboa.pt; 2 School of Chemistry and Chemical Engineering, Queen’s University Belfast David Keir Building, Stranmillis Road, Belfast, Northern Ireland BT9 5AG, UK; goodrich@qub.ac.uk

**Keywords:** carbon dioxide conversion, cyclic carbonate, recyclable catalyst, iron C-scorpionate, synergistic catalysis

## Abstract

The production of cyclic carbonates from CO_2_ cycloaddition to epoxides, using the C-scorpionate iron(II) complex [FeCl_2_{κ^3^-HC(pz)_3_}] (pz = 1*H*-pyrazol-1-yl) as a catalyst, is achieved in excellent yields (up to 98%) in a tailor-made ionic liquid (IL) medium under mild conditions (80 °C; 1–8 bar). A favorable synergistic catalytic effect was found in the [FeCl_2_{κ^3^-HC(pz)_3_}]/IL system. Notably, in addition to exhibiting remarkable activity, the catalyst is stable during ten consecutive cycles, the first decrease (11%) on the cyclic carbonate yield being observed during the 11th cycle. The use of C-scorpionate complexes in ionic liquids to afford cyclic carbonates is presented herein for the first time.

## 1. Introduction

Over the last few decades, it has become clear that sharply increasing anthropogenic CO_2_ emissions is affecting the climate stability of the biosphere [1]. Therefore, a realistic transition from fossil carbon usage to alternative raw materials and commodities based on recycled carbon (CO_2_) is imperative. Since current carbon capture and storage (CCS) technologies are able to capture up to 90% of the CO_2_ pro­duced [2], rendering it available in vast quantities and with satisfactory purity, a sustainable solution would entail its conversion into useful value-added commodity chemicals [3,4,5,6,7,8,9,10,11]. Among the possible strategies to use captured CO_2_, its catalytic reaction with epoxides to produce cyclic carbonates is one of the most promising applications as a renewable carbon source. The interest in cyclic carbonates is driven by their wide range of chemical and technological applications. To date, a considerable number of catalytic systems have been developed (either metal or organocatalysts) for the cycloaddition of CO_2_ and epoxides [3,4,5,6,7,8,9,10,12,13,14,15,16,17,18,19,20,21,22,23,24]. However, further improvements are needed, in particular, in (i) controlling the selectivity (to impair polycarbonates formation); (ii) achieving suitable catalytic activities for less reactive substrates (e.g., sterically hindered and internal epoxides); (iii) searching for milder efficient reaction conditions (high temperatures and pressures are ultimately associated with additional, indirect CO_2_ emissions, limiting their value from a technological standpoint); and (iv) finding active and selective catalysts able to be recycled and reused in consecutive cycles.

**Figure 1:**
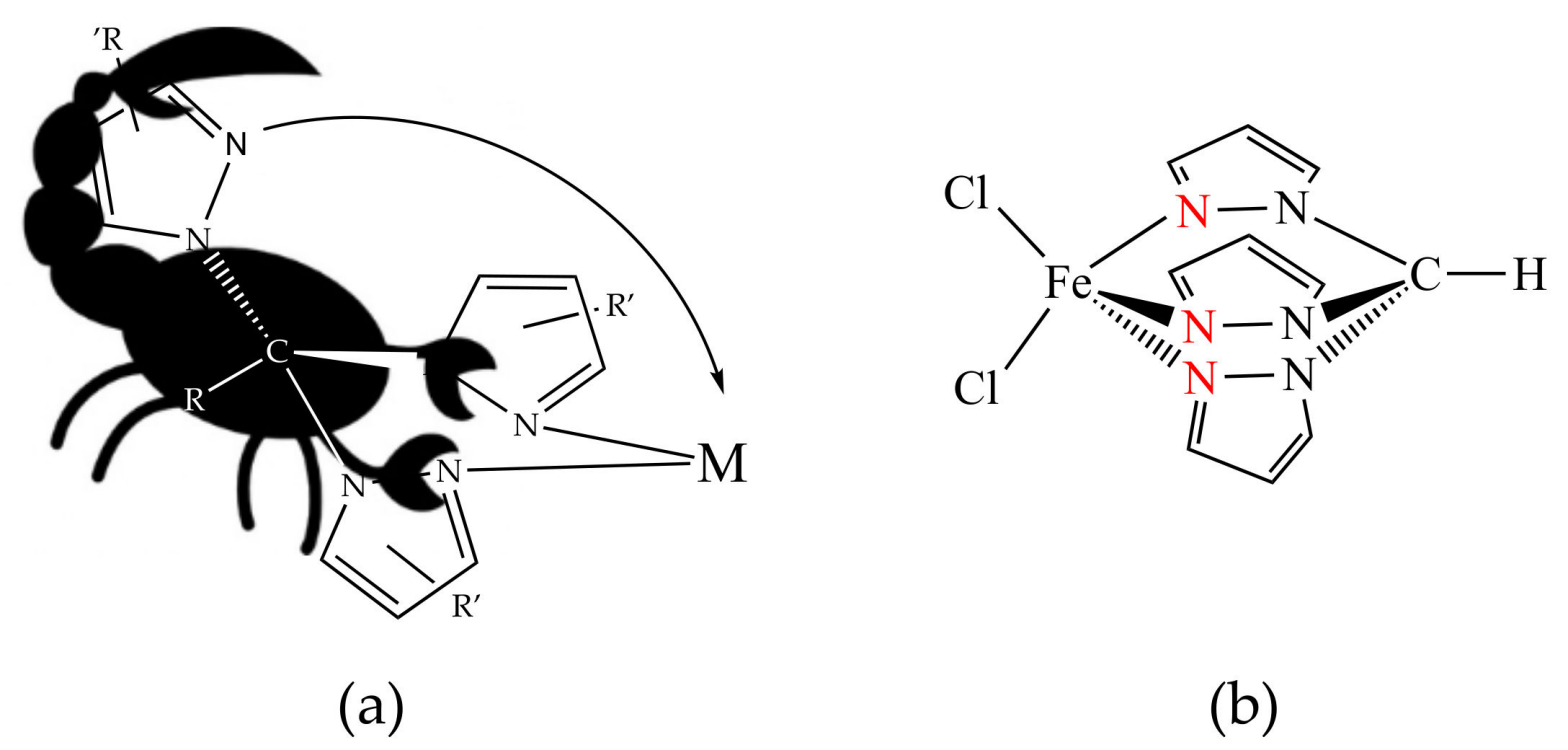
(**a**) Analogy between a C-scorpionate coordination to a metal center (M) and the attack of a scorpion on its prey. (**b**) Structure of the dichloro hydrotris(1*H*-pyrazol-1-yl)-methane Fe(II) complex [FeCl_2_{κ^3^-HC(pz)_3_}] (pz = 1*H*-pyrazol-1-yl).

Tripodal nitrogen poly(1*H*-pyrazol-1-yl)-methane scorpionate ligands, [R_(4−n)_C(R’pz)_n_] (pz = 1*H*-pyrazol-1-yl), also known as C-scorpionates (analogy with a scorpion; see Figure 1a), are one of the most versatile classes of ligands in coordination chemistry. They are able to (i) stabilize transition metals in a wide range of oxidation states, (ii) combine the tripodal architecture needed for efficient three-point biding with a chelating coordination site, leaving up to three sites to other co-ordinations; and (iii) provide a degree of steric bulk, avoiding, e.g., dimerization reactions. Variation of the substituents at different positions on the pyrazolyl rings, or at the at the methine carbon atom, leads to a range of steric and electronic effects, with this tunability being one of the most important advantages of poly(1*H*-pyrazol-1-yl)-methane ligands [25,26,27].

C-scorpionate complexes have found a wide range of applications in bioinorganic chemistry [26,28,29,30], in particular in metalloenzyme modelling studies mimicking histidine nitrogen coordination by pyrazole to the metal ion binding sites at Cu proteins (e.g., hemocyanin, ascorbate oxidase, and superoxide dismutase), Fe proteins (hemerythrin), or Mn proteins (superoxide dismutase) [31]. 

The ability of these ligands to readily modify their scaffold, either at the pyrazolyl rings or/and at the methinic carbon atom, to tailor electronic, steric, and coordination properties as desired for a particular application, could provide unique catalytic effects towards the application of CO_2_ and epoxides as raw materials for the sustainable synthesis of cyclic carbonates.

In this work, the above cycloaddition reaction issues, in particular iv), finding active and selective catalysts able to be recycled and reused in consecutive cycles, are addressed by using the bio-inspired C-homoscorpionate Fe(II) catalyst [FeCl_2_{κ^3^-HC(pz)_3_}] (pz = 1*H*-pyrazol-1-yl) [25,26,27] in tailor-made cheap ionic liquid media [32]. The selected [FeCl_2_{κ^3^-HC(pz)_3_}] complex (Figure 1b) exhibits a tetragonal pyramid coordination polyhedron with one of the donor N pyrazolyl atoms at the axial position and where the iron atom has one empty coordination site that could be easily occupied by a new substrate [33], such as an epoxide. Moreover, it is easy (one step) to synthesize [34], in water, at r.t., from the available and cheap iron salt and the simplest ligand of the tris(1*H*-pyrazol-1-yl)methane carbon scorpionate class, hydrotris(1*H*-pyrazol-1-yl)-methane, HC(pz)_3_. To our knowledge, to date, this class of compounds has not been tested as a catalyst for cyclic carbonate synthesis.

## 2. Results and Discussion

**Figure 2:**
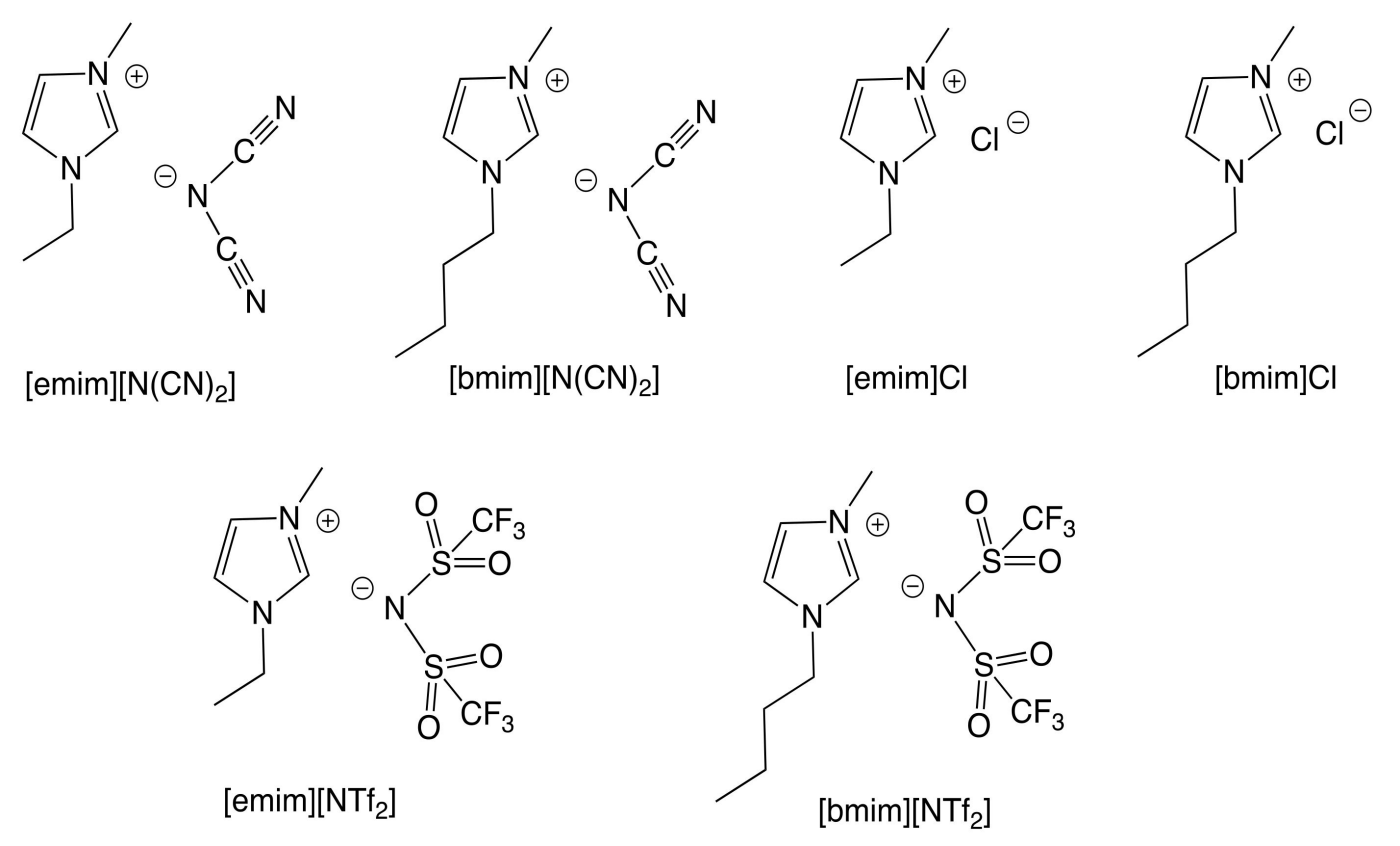
Room-temperature ionic liquids used in this work.

**Scheme 1:**
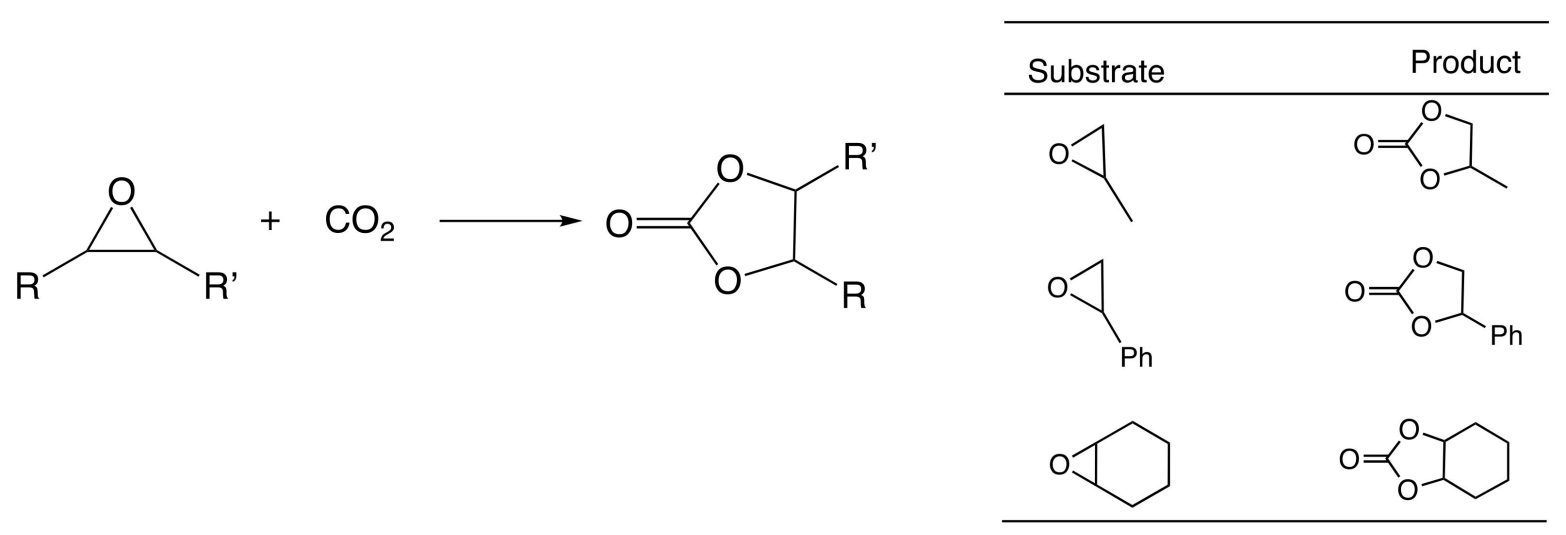
Selective cycloaddition of CO_2_ to epoxides.

At first, the knowledge [9,14,15,16,17] that some types of ionic liquids are able to catalyze the cycloaddition of CO_2_ to epoxides prompted us to perform a series of experiments with the green, commercially available and affordable ionic liquids (IL) depicted in Figure 2 and selected model epoxides (Scheme 1). In addition to the typical propylene and styrene oxides (bearing an alkyl or phenyl group, respectively), the very challenging (internal epoxide) cyclohexene oxide was also chosen.

The starting reaction conditions were selected in view of the reported [35] cycloaddition reaction to cyclohexene oxide substrate. Then, the reaction parameters (e.g., time, temperature, CO_2_ pressure, IL to substrate ratio) were optimized (see experimental section) but the obtained carbonate yields under such conditions (epoxide (5 mmol), [^t^Bu_4_N]Br (0.3%mol vs. epoxide), IL (8.3–18.8 mol), CO_2_ (8 bar), 24 h, 80 °C) were very low (up to 5%, for [bmim][N(CN)_2_]), even in the presence of promoting [Bu_4_N]Br [36,37] (which apparently requires higher temperatures and CO_2_ pressures to operate as a catalyst). Note that the presence of the ionic liquid allowed us to use a lower amount of [^t^Bu_4_N]Br than the level usually reported in the literature [6,38,39] as being required to achieve high epoxide conversions. Nevertheless, in all experiments, only a single reaction product, the corresponding carbonate, was identified, as shown in Scheme 1.

**Figure 3:**
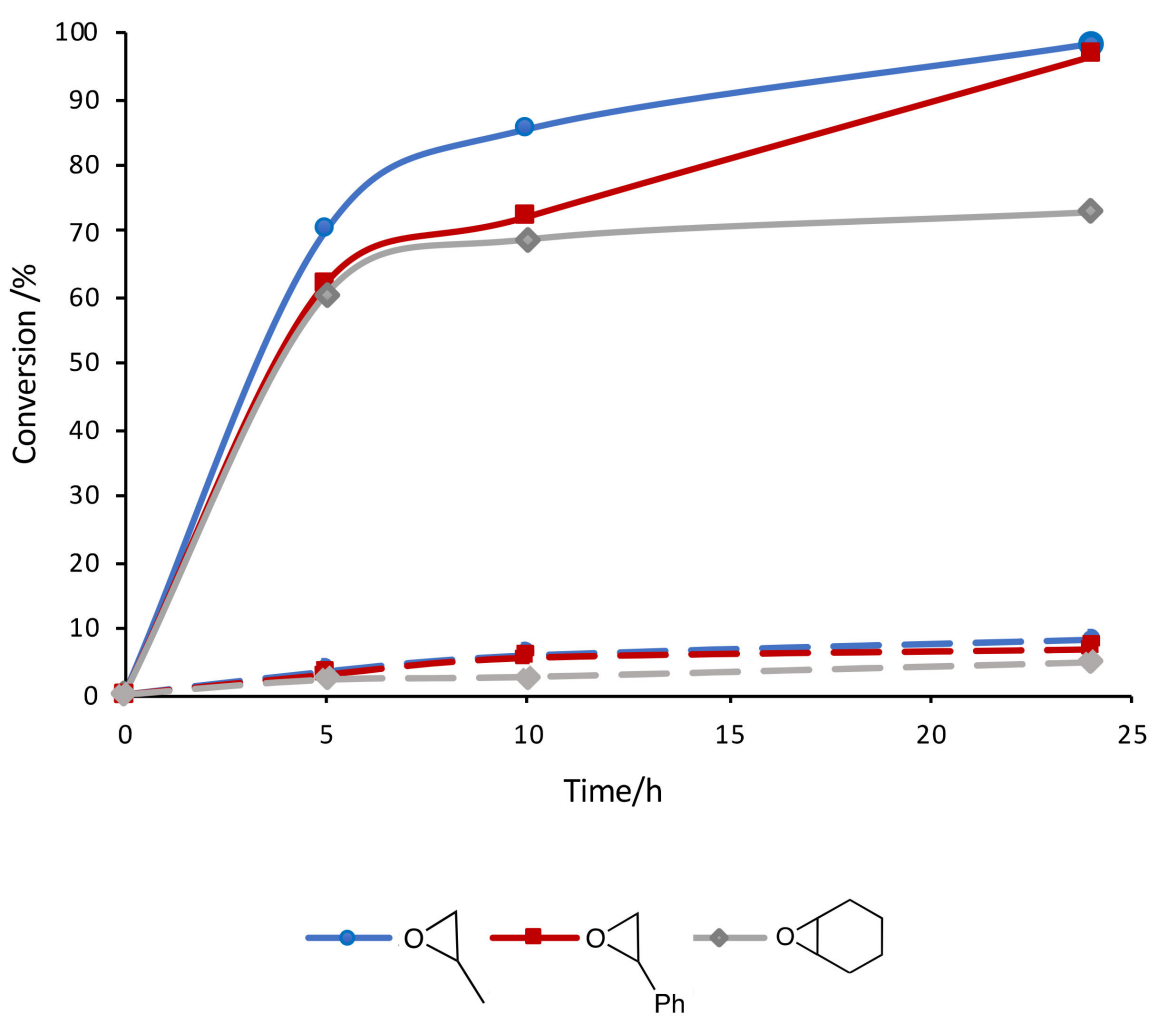
Kinetic profile of the cycloaddition of CO_2_ to different epoxides catalyzed by the [FeCl_2_{κ^3^-HC(pz)_3_}]/IL system (solid lines, in [bmim][N(CN)_2_]; dashed lines, in THF).

The IL [bmim][N(CN)_2_] is known to interact with the C-homoscorpionate iron(II) complex [FeCl_2_{κ^3^-HC(pz)_3_}] (pz = 1H-pyrazol-1-yl) and a favorable catalytic synergistic effect was reported [33] for other reactions. Iron catalysts in CO_2_ chemistry have been reported for CO_2_ reduction and hydroformylation reactions, but their use in CO_2_/epoxide chemistry has been less extensively explored. Therefore, herein the above system was tested as a catalyst for the synthesis of cyclic carbonates under the above optimal conditions. The obtained results are presented in Table 1. For propylene and styrene oxide substrates, excellent yields (up to 98.3%, in [bmim][N(CN)_2_]), entry 2, Table 1) were achieved for the corresponding cyclic carbonates concomitant with a selectivity of 100%. Even the less reactive cyclohexene oxide was successfully converted, attaining good carbonate yields (up to 72.7%, in [bmim][N(CN)_2_]), entry 14, Table 1). [FeCl_2_{κ^3^-HC(pz)_3_}] was added to the reaction medium within a 0.3–0.8% mol vs. epoxide range, exhibiting its best performance at a concentration of 0.5% mol vs. epoxide. The increase up to 0.8% mol vs. epoxide did not lead to a significant improvement in the products yields, and therefore the lowest 0.5% mol vs. epoxide was selected. The kinetic profiles are depicted in Figure 3.

All three substrates follow the trend of achieving conversions depending in the IL anions in the following order: N(CN)_2_ > Cl > NTf_2_. Ionic liquids with Cl^−^ and [NTf_2_]^−^ anions exhibit significantly higher viscosity ([emim]Cl, 44 cP; [bmim]Cl 55 cP; [emim][NTf_2_], 100 cP; [emim][NTf_2_], 190 cP [40]) than ILs bearing [N(CN)_2_]^−^ counterions ([emim][N(CN)_2_], 15 cP; [emim][N(CN)_2_], 33 cP [40]) as a result of intra and intermolecular hydrogen bonding and van der Waals interactions [41]. Since a high viscosity directly affects the mass transfer of a reaction, and vice versa [42], the viscosity imparted by the anion of the ionic liquid appears to be an important parameter in the study of the catalytic performance of the C-scorpionate Fe(II) complex in such media. The observed catalyst activity could also be linked to the relative coordination ability of these anions to the metal center; however, this clearly needs further investigation.

The ionic liquids’ catalytic performance was also studied, and the results for the conversion of styrene epoxide into styrene carbonate are presented in Table 2. Under the reaction conditions used, the ionic liquids of this study, per se, did not exhibit catalytic activity for the cycloaddition of carbon dioxide and epoxides.

It is worth mentioning that the [FeCl_2_{κ^3^-HC(pz)_3_}] complex by itself, although maintaining the selectivity found for the [FeCl_2_{κ^3^-HC(pz)_3_}]/IL system, exhibited a much worse catalytic performance, leading to carbonate yields of up to 6.5% (Table 3).

Thus, the synergistic catalysis found by combining [FeCl_2_{κ^3^-HC(pz)_3_}] with the appropriate IL (preferably [bmim][N(CN)_2_]) significantly improved the efficiency of the present carbonate synthetic process. Conversely, poor yields were observed in the molecular solvent THF, highlighting the benefits of conducting the process in an IL media.

The [FeCl_2_{κ^3^-HC(pz)_3_}]/IL catalytic system exhibits better performance and/or requires milder reaction conditions than several other catalysts, including organocatalysts and iron-based catalysts previously found in the literature [6,38,43,44,45,46]. Table 4 presents a comparison of some of these catalytic systems. 

For example, Park et al., [43] used alkylmethylimidazolium-based ionic liquids, bearing the anions Cl^−^, [BF_4_]^−^ or [PF_6_]^–^, as catalysts for the cycloaddition of CO_2_ (9.6 bar) to allyl glycidyl ether, which took 48 h at 100 °C to generate the five-membered cyclic carbonate in good yields (entry 1, Table 4). On the other hand, the binary catalytic system presented in entry 2 of Table 4, although operating at mild conditions (25 °C, 1.01 bar of CO_2_ for 24 h) led to moderate carbonate yields [44]. 

Jing et al. [45] reported an iron porphyrin complex as a catalyst for the coupling of propylene oxide and CO_2_, yielding a propylene oxide conversion of only 10% after 3 h (entry 3, Table 4). Recently, Jones et al., [46] used a series of air-stable iron(III) acetate complexes bearing salan, salen, or salalen ligands for the coupling of CO_2_ and the challenging cyclohexene oxide substrate in the presence of tetrabutylammonium chloride (TBAC) as co-catalyst and under solvent-free conditions, at 80 °C and 10 bar CO_2_. The cis-cyclohexene carbonate was selectively formed as the exclusive product (entry 4, Table 4). Thus, under quite similar conditions, our C-scorpionate Fe(II)/IL catalyst exhibited superior performance, leading to higher carbonate yields. However, although leading to significantly higher conversion values of propylene, styrene, and cyclohexene oxides (98.3, 94.6, and 72.7%, entries 2, 8, and 14 of Table 1, respectively) than those obtained with amino-bis(phenolate) iron (II) complexes (74, 31 and 9%, respectively) [10], the later catalytic system led to higher turnover number (TON) or turnover frequency (TOF) values (2960, 1240, and 364, respectively, for propylene, styrene, and cyclohexene oxides) in comparison to ours (197, 193 and 145, entries 2, 8, and 14 of Table 1 respectively). A similar behavior was found for thioether-tiophenolate bimetallic iron(III) complexes [9], leading to conversions of propylene oxide up to 50% and reaching the remarkable TOF value of 4990 h^−1^. 

Notably, none of the above studies reported the recovery or reusability of the used iron catalysts [6,9,12,45,46].

**Figure 4:**
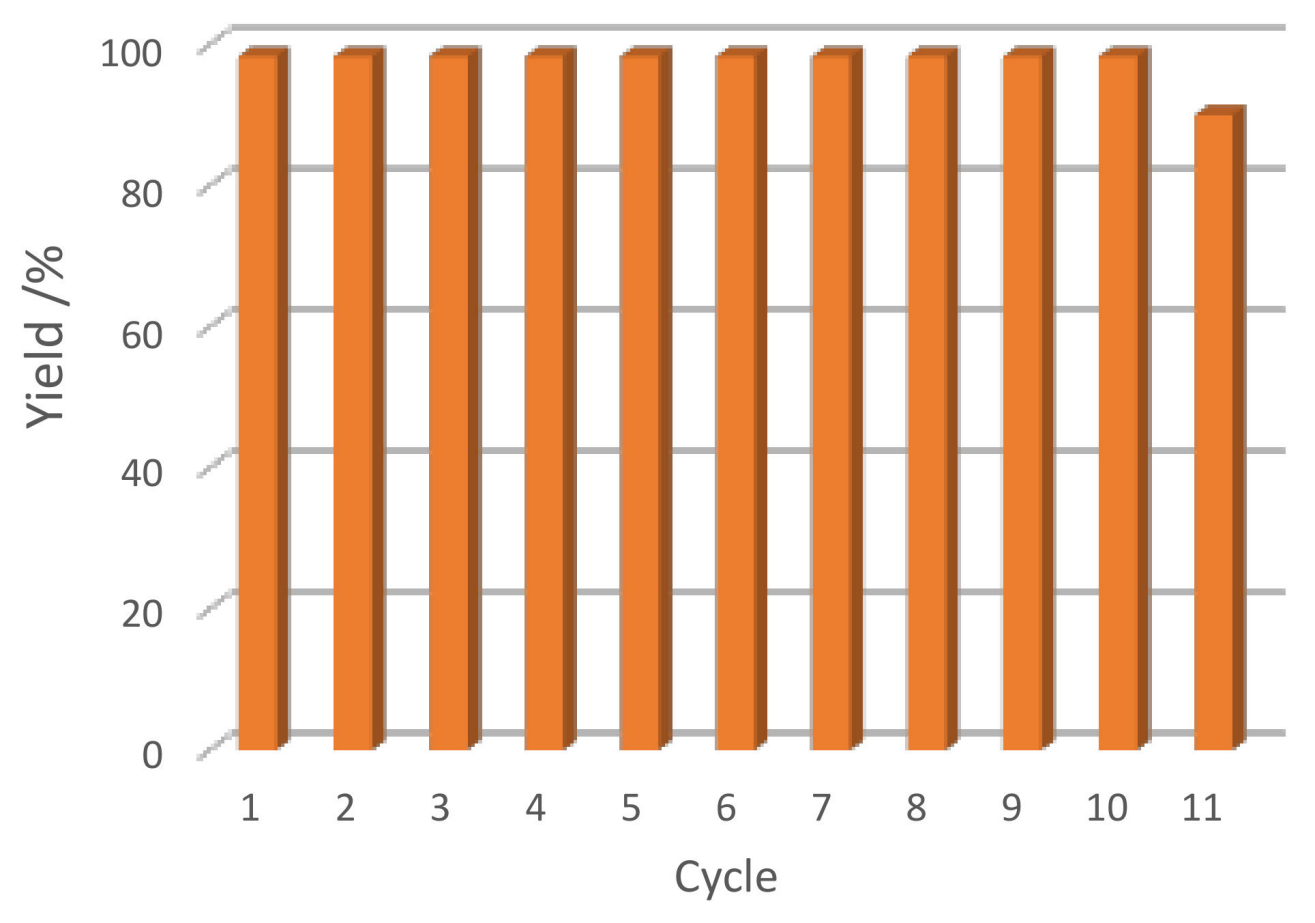
Effect of the number of catalytic cycles on the yield of the cyclic carbonate obtained by cycloaddition of CO_2_ to propylene oxide catalyzed by [FeCl_2_{κ^3^-HC(pz)_3_}] in [bmim][N(CN)_2_].

Herein, the stability of the [FeCl_2_{κ^3^-HC(pz)_3_}]/IL catalytic system is a relevant advantage that allowed it to be recycled and reused at least for ten consecutive cycles without losing its initial activity (see Figure 4 for [bmim][N(CN)_2_]).

Remarkably, our system of producing cyclic carbonates presents improvements relative to previously reported ones, that can overcome the undesirable low TON or TOF values. It can be considered sustainable and economical as its activity was almost constant after recycling at least ten times.

**Figure 5:**
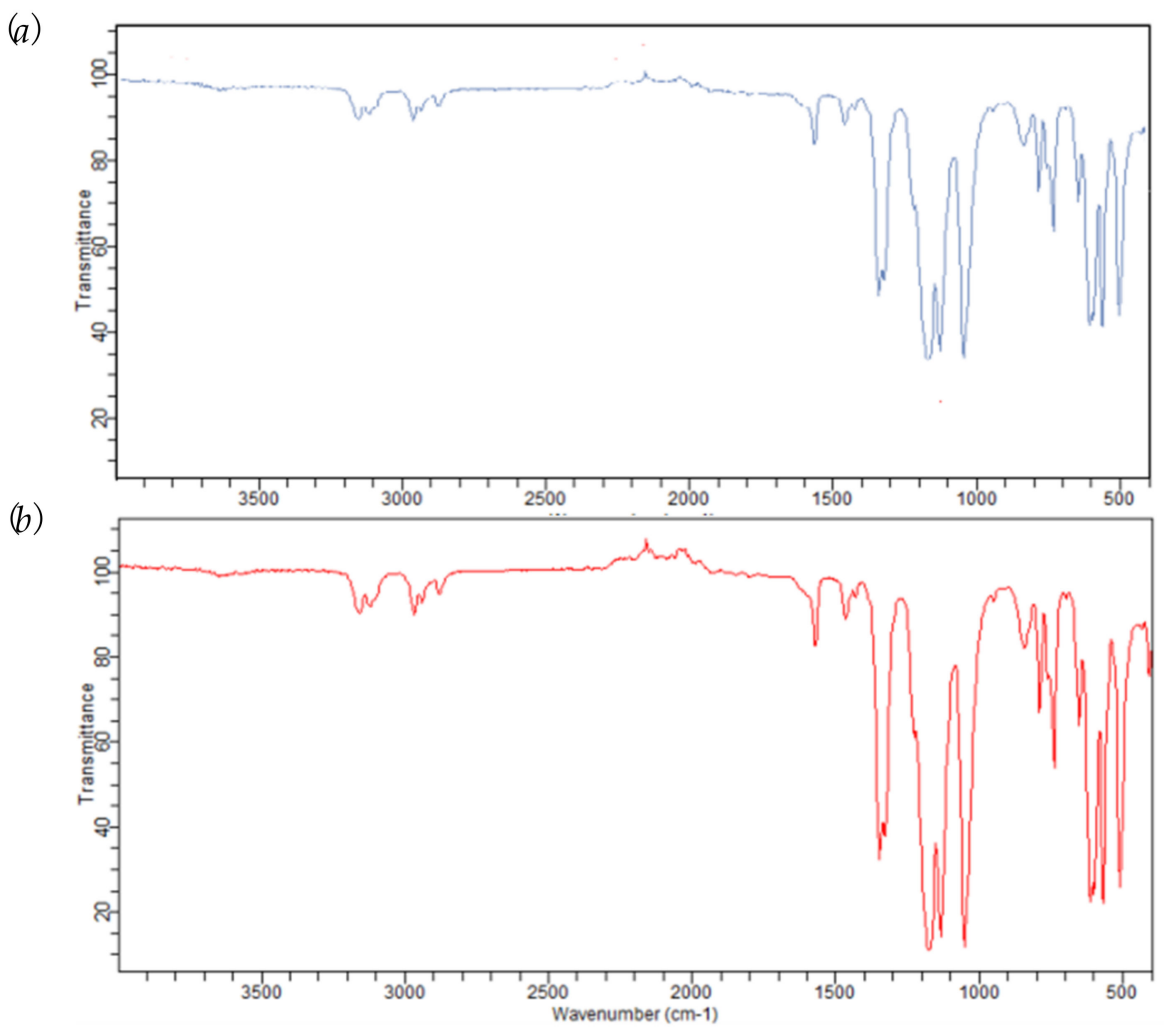
Infrared spectra in the 500–4000 cm^−1^ range of (**a**) [FeCl_2_{κ^3^-HC(pz)_3_}]/[bmim][NTf_2_] and (**b**) FeCl_2_{κ^3^-HC(pz)_3_}]/[bmim][NTf_2_] after the 4th catalytic run.

The IR spectra in the 4000–500 cm^−1^ range of FeCl_2_{κ^3^-HC(pz)_3_}]/IL before and after the four consecutive catalytic cycles matched (compare Figure 5a,b), proving the stability of the C-scorpionate complex in the reaction ionic liquid medium.

Previous works on the synthesis of cyclic carbonates from CO_2_ and epoxides suggested the parallel requirement of both Lewis-base activation of the CO_2_ and Lewis-acid-activation of the epoxide [6,38]. Thus, it is likely that the C-scorpionate Fe(II) center could serve as a Lewis acid for epoxide coordination and activation (where the scorpionate ligand has a key role), whereas the ionic liquid would activate CO_2_ and combined with TBAB (added as cocatalyst) would promote bromide (and chlorine or DCA) anions to act as a nucleophile, thereby promoting the ring opening. This would be followed by the ring opening of the coordinated epoxide and consequent intramolecular ring closing, releasing the cyclic carbonate and recovering the catalytic system. 

To best of our knowledge, reports regarding possible reaction mechanisms for the cycloaddition of CO_2_ to epoxides are scarce. DFT calculations for propylene oxide substrate in the absence and presence of alkylmethylimidazolium chloride ([C_n_mim]Cl, n = 2, 4, or 6) ionic liquids performed by H. Sun et al. [47] suggested that cycloaddition is a multipath reaction that could proceed through two and five (or more) possible routes in the absence or presence of [C_n_mim]Cl, respectively. In all the cases considered, the rate-determining steps involve the ring opening of the epoxide. The study also demonstrated that there are cooperative actions of the cation and anion of the ionic liquid which stabilize intermediates as well as transition states through hydrogen-bonding interaction, thus facilitating the ring opening of the epoxide.

## 3. Materials and Methods

The reagents were purchased from Aldrich (St. Louis, MO, USA) and used without further purification. Carbon dioxide gas of 99.99% purity was used. Hydrotris(1*H*-pyrazol-1-yl)methane, HC(pz)_3_, was synthesized according to the literature method [48,49]. The C-scorpionate complex [FeCl_2_{κ^3^-HC(pz)_3_}] was prepared according to the literature [34] and characterized by the conventional techniques. 

The cycloaddition reactions of CO_2_ and epoxide were carried out in a stainless-steel autoclave (16.0 cm^3^) equipped with a stirrer and temperature control system. The selected epoxide (2.0–10.0 mmol), THF (2.5 cm^3^), or the chosen ionic liquid (8.3–18.8 mol), tetra-n-butylammonium bromide, [Bu_4_N]Br (TBAB, 0.3% mol vs. epoxide) and, when used, [FeCl_2_{κ^3^-HC(pz)_3_}] (0.3–0.8% mol vs. epoxide), were added to the autoclave. Then, the reactor was purged twice and the CO_2_ selected pressure (1–8 bar) was charged in the reactor at r.t. The reaction was carried out at temperature within the 30–100 °C range, under autogenous conditions for the desired time (up to 24 h) with continuous stirring (600 rpm). The autoclave was cooled to r.t., the excess of pressure released, and the product(s) analyzed by ^1^H-NMR spectroscopy (Bruker 400 UltraShield^TM^ spectrometers, Rheinstetten, Germany); ^1^H chemical shifts *δ* expressed in ppm relative to SiMe_4_) after extraction from the IL media. The product quantification was performed by applying the internal standard method using CDCl_3_ (400 μL, used both as solvent and internal standard).

Catalyst recyclability in the IL medium under the optimal experimental reaction conditions was investigated. Each cycle was initiated after the preceding one upon the addition of new typical portions of all other reagents. After the completion of each run, the organics were extracted for analysis (see above), and the IL which contained the (dissolved) catalyst was washed several times with ether and dried in vacuo overnight at 70 °C. The stability of the catalyst was verified by comparison of the FTIR-ATR spectra (in a Bruker Vertex 40 Raman/IR spectrometer, Rheinstetten, Germany); of the mixture ([FeCl_2_{κ^3^-HC(pz)_3_}]/IL) before and after each catalytic run.

## 4. Conclusions

In conclusion, the use of scorpionate-based complexes in an IL media can provide high selectivity to cyclic carbonates in moderate to high yields under mild conditions. In particular, the cycloaddition of carbon dioxide and an epoxide in the presence of the C-homoscorpionate Fe(II) catalyst [FeCl_2_{κ^3^-HC(pz)_3_}] (pz = 1*H*-pyrazol-1-yl) in tailor-made cheap ionic liquid media such as [bmim][N(CN)_2_] and under mild conditions (80 °C; 1–8 bar) selectively yields up to 98% of the corresponding cyclic carbonate. In addition to a remarkable activity, the catalyst exhibits superior stability during ten consecutive catalytic cycles, a clear advantage relative to previously reported catalytic systems.

Future work is currently ongoing to extend the range of substrates and investigate the accelerated yields observed in IL compared to a molecular solvent, as well as to establish the corresponding reactional mechanisms. 

## Figures and Tables

**Table 1:** Selected data ^a^ for the cycloaddition of CO_2_ to epoxides catalyzed by [FeCl_2_{κ^3^-HC(pz)_3_}] in different ionic liquid (IL) media.

Entry	Ionic Liquid	Epoxide	Product	Yield/% ^b^	TON ^c^	TOF/h^−1 d^
1	[emim][N(CN)_2_]	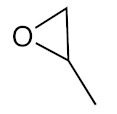	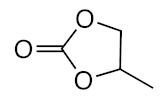	96.7	193	8
2	[bmim][N(CN)_2_]			98.3	197	8
3	[emim]Cl			81.6	163	7
4	[bmim]Cl			90.0	180	8
5	[emim][NTf_2_]			72.8	146	6
6	[bmim][NTf_2_]			78.7	157	7
7	[emim][N(CN)_2_]	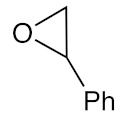	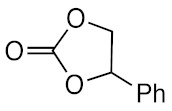	92.3	185	8
8	[bmim][N(CN)_2_]			96.4	193	8
9	[emim]Cl			77.4	155	6
10	[bmim]Cl			88.7	177	7
11	[emim][NTf_2_]			61.9	124	5
12	[bmim][NTf_2_]			72.6	145	6
13	[emim][N(CN)_2_]	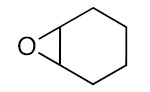	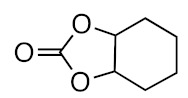	69.3	139	6
14	[bmim][N(CN)_2_]			72.7	145	6
15	[emim]Cl			60.7	121	5
16	[bmim]Cl			69.3	139	6
17	[emim][NTf_2_]			40.7	81	3
18	[bmim][NTf_2_]			60.9	122	5

^a^ Reaction conditions: epoxide (5 mmol), [Bu_4_N]Br (0.3%mol vs. epoxide), [FeCl_2_{κ^3^-HC(pz)_3_}] (0.5%mol vs. epoxide), IL (8.3–18.8 mol), CO_2_ (8 bar), 24 h, 80 °C. ^b^ Yield determined by ^1^H-NMR (internal standard method). ^c^ Turnover number (moles of cyclic carbonate per mol of [FeCl_2_{κ^3^-HC(pz)_3_}] catalyst). ^d^ Turnover frequency (turnover number per hour).

**Table 2:** Cycloaddition ^a^ of CO_2_ to styrene epoxide in different ionic liquids.

Entry	Ionic Liquid	Styrene Carbonate Yield/% ^b^
1	[emim][N(CN)_2_]	0.3
2	[bmim][N(CN)_2_]	4.9
3	[emim][Cl]	0.5
4	[bmim][Cl]	1.6
5	[emim][NTf_2_]	0.1
6	[bmim][NTf_2_]	0.3

^a^ Reaction conditions: styrene epoxide (5 mmol), [Bu_4_N]Br (0.3% mol vs. epoxide), IL (2.5 mL), CO_2_ (8 bar), 24 h, 80 °C. ^b^ Yield determined by ^1^H NMR (internal standard method).

**Table 3:** Selected data ^a^ for the cycloaddition of CO_2_ to epoxides catalyzed by [FeCl_2_{κ^3^-HC(pz)_3_}] in THF.

Entry	Epoxide	Product	Yield/% ^b^
1	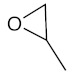	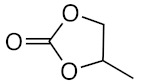	6.5
2	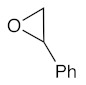	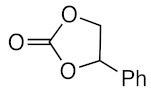	6.1
3	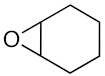	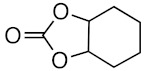	5.3

^a^ Reaction conditions: epoxide (5 mmol), [Bu_4_N]Br (0.3% mol vs. epoxide), FeCl_2_{κ^3^-HC(pz)_3_}] (0.5%mol vs. epoxide), THF (2.5 mL), CO_2_ (8 bar), 24 h, 80 °C. ^b^ Yield determined by ^1^H NMR (internal standard method).

**Table 4:** Cycloaddition of CO_2_ to epoxides catalyzed by different classes and types of homogeneous catalysts.

Entry	Catalytic System	Epoxide	Carbonate Yield/%	Reference
1	1-alkyl-3-methylimidazolium chloride	allyl glycidyl ether	81	[43]
2	ionic liquid/2,6-pyridinedimethanol/TBAB	epichlorohydrin	67	[44]
3	porphyrin iron complex	propylene oxide	10	[45]
4	iron(III)–acetate complexes/TBAC	cyclohexene oxide	up to 66	[46]
